# Endosomes Derived from Clathrin-Independent Endocytosis Serve as Precursors for Endothelial Lumen Formation

**DOI:** 10.1371/journal.pone.0081987

**Published:** 2013-11-25

**Authors:** Natalie Porat-Shliom, Roberto Weigert, Julie G. Donaldson

**Affiliations:** 1 Cell Biology and Physiology Center, National Heart, Lung and Blood Institute, National Institutes of Health, Bethesda, Maryland, United States of America; 2 Intracellular Membrane Trafficking Unit, Oral and Pharyngeal Cancer Branch, National Institute of Dental and Craniofacial Research, National Institutes of Health, Bethesda, Maryland, United States of America; Cambridge University, United Kingdom

## Abstract

Clathrin-independent endocytosis (CIE) is a form of bulk plasma membrane (PM) endocytosis that allows cells to sample and evaluate PM composition. Once in endosomes, the internalized proteins and lipids can be recycled back to the PM or delivered to lysosomes for degradation. Endosomes arising from CIE contain lipid and signaling molecules suggesting that they might be involved in important biological processes. During vasculogenesis, new blood vessels are formed from precursor cells in a process involving internalization and accumulation of endocytic vesicles. Here, we found that CIE has a role in endothelial lumen formation. Specifically, we found that human vascular endothelial cells (HUVECs) utilize CIE for internalization of distinct cargo molecules and that in three-dimensional cultures CIE membranes are delivered to the newly formed lumen.

## Introduction

Endocytosis is viewed as a process by which cells down-regulate plasma membrane (PM) proteins. However, in recent years, it has become clear that endocytosis and the subsequent trafficking of endosomal membranes have crucial roles in multiple biological processes including cell migration, division, signaling, transcription, and actin dynamics [1-3]. There are two general types of endocytosis: those dependent upon clathrin and its adaptor proteins and those that are independent of clathrin coats [4,5]. In clathrin-mediated endocytosis (CME) cargo proteins are efficiently endocytosed by virtue of specific cytoplasmic sequences that bind to the adaptor proteins. Clathrin-independent endocytosis (CIE), on the other hand, brings in cargo proteins that lack these specific sequences and hence is often thought of as a method to internalize bulk PM cargo proteins. Once inside the cell, the vesicle carriers that arise from either CME or CIE fuse with the early sorting endosome [6,7]. From here, cargo proteins can be directed to the lysosome for degradation or recycled back to the PM using a complex network of endosomal membranes. A wide variety of endogenous cargo proteins enter cells through a distinct form of CIE that is cholesterol-dependent, dynamin-independent and associated with Arf6 [6-20]. Aside from membrane proteins, many signaling molecules associate with and signal from these endosomes that arise from CIE including Arf6, Arf1, H-Ras, Erk and Src [3,21-25]. Furthermore, components of the PM, such as the lipids, actin and cholesterol, are transported via this pathway suggesting additional physiological roles for these non-canonical membrane carriers [3]. 

Roles for endocytic uptake and recycling have been described in diverse processes critical for the establishment of cell polarity such as budding in yeast and cell migration in mammalian cells [26-30]. Morphogenesis of the endothelial tube is an additional process where polarity is established and is essential for the development of a functional vertebrate circulatory system. Multiple models have been proposed for endothelial lumen formation including ‘cell hollowing’, ‘cord hollowing’, ‘budding’, ‘membrane invagination’ and ‘lumen ensheathment’ [31-36]. Although these different models seem very diverse, it is likely that in different organisms or under various physiological conditions the endothelial lumen will form through distinct mechanisms. *In vitro* and *in vivo* evidence support a model by which endothelial lumens are formed by a mechanism referred to as cell hollowing [37,38]. According to this model, the vascular lumen is initiated in a single cell by the internalization of pinocytic vesicles through an undefined mechanism, which then fuses to form an enlarged apical vacuole [32,39]. Recent studies have implicated Arf6 in epithelial morphogenic processes [40,41], which led us to speculate that CIE might contribute to the production and maintenance of cell polarity and more specifically in the process of endothelial lumen formation. In the present study we show that Human Umbilical Vein Endothelial Cells (HUVECs) internalize CIE cargo proteins in distinct endosomes and that when grown in a collagen matrix, these endosomes deliver membranes to the newly formed lumen. 

## Results

### CME and CIE in HUVECs

**Figure 1:**
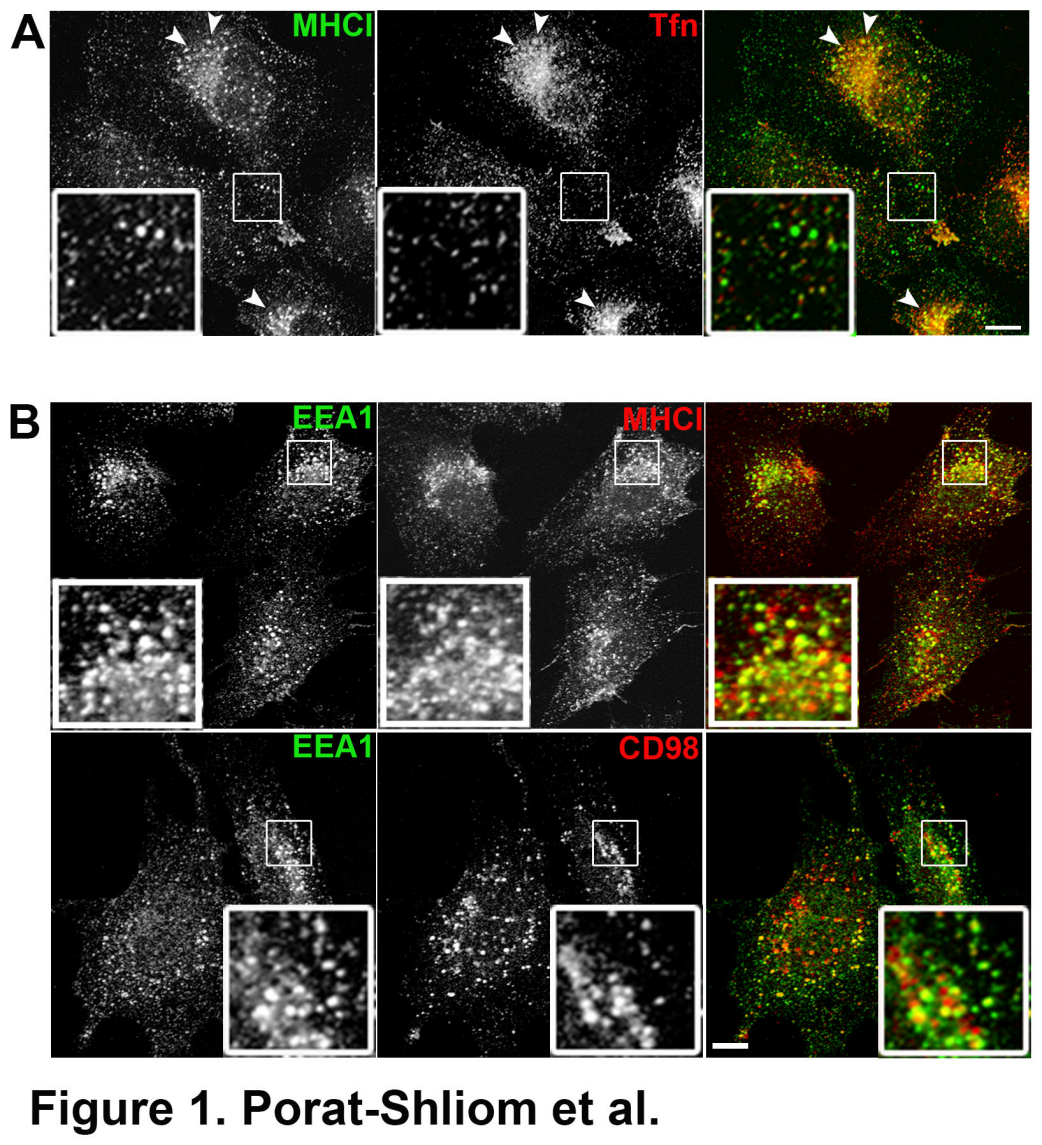
CIE and CME occur in HUVECs. Cells were incubated with an antibody against MHCI and 594-labeled transferrin for 30 min. Internalized MHCI was visualized with 488-labeled secondary antibodies. MHCI and transferrin (Tfn) internalized in distinct carriers (inset) but were observed co-localized in the perinuclear region (arrowheads). (B) Internalization of MHCI antibody (upper panel) or CD98 antibody (lower panel) for 30 min was performed in HUVECs. After fixation, cells were labeled with the EEA1 antibody as described in Materials and Methods. Internalized MHCI and CD98 both reached an EEA1 positive endosome. Bar, 10 μm.

CIE has been observed in many human cell lines [5,9]. To establish whether CIE has a role in endothelial lumen formation, we first characterized CIE cargo trafficking in HUVECs and compared it to that of clathrin cargo proteins. To do so, we performed an internalization assay in which monoclonal antibodies against the major histocompatibility complex Class I molecule (MHCI) and fluorescently labeled transferrin (Tfn) were added to the media to label and follow clathrin-independent and clathrin-mediated cargo molecules, respectively [6]. After 30 min, most of the internalized MHCI was localized in endosomes that lacked transferrin whereas only a sub-population of them co-localized with transferrin in the juxtanuclear region (Figure 1A arrowheads). Similar results were obtained with other CIE cargo molecules (CD98 or CD147) with labeled transferrin (data not shown). Previous studies showed that CIE cargo proteins might have different trafficking itineraries in various cell types [9,42]. Indeed, MHCI is sorted into recycling tubules from an endosomal compartment containing transferrin and associated with early endosomal antigen 1 (EEA1) (sorting endosomes), while CD98 and CD147 by-pass this compartment and directly enter the recycling tubes [9,42]. We found that in HUVECs both MHCI and CD98 reached an early endosomal compartment positive for EEA1 (Figure 1B) although the extent of co-localization between MHCI and EEA1 was higher than that of CD98 and EEA1 (~47% for MHCI and ~20% for CD98 p value < 0.02; Figure S1A). Furthermore, after 1 h internalization of antibodies to MHCI and CD98 followed by 14 h incubation in media containing ammonium chloride (NH_4_Cl) to inhibit lysosomal degradation, both antibodies accumulated in a LAMP1 positive compartment (Figure S1B). This suggests that both MHCI and CD98 utilize the same incoming route in HUVECs. Similar results were also obtained with CD147 (data not shown).

**Figure 2:**
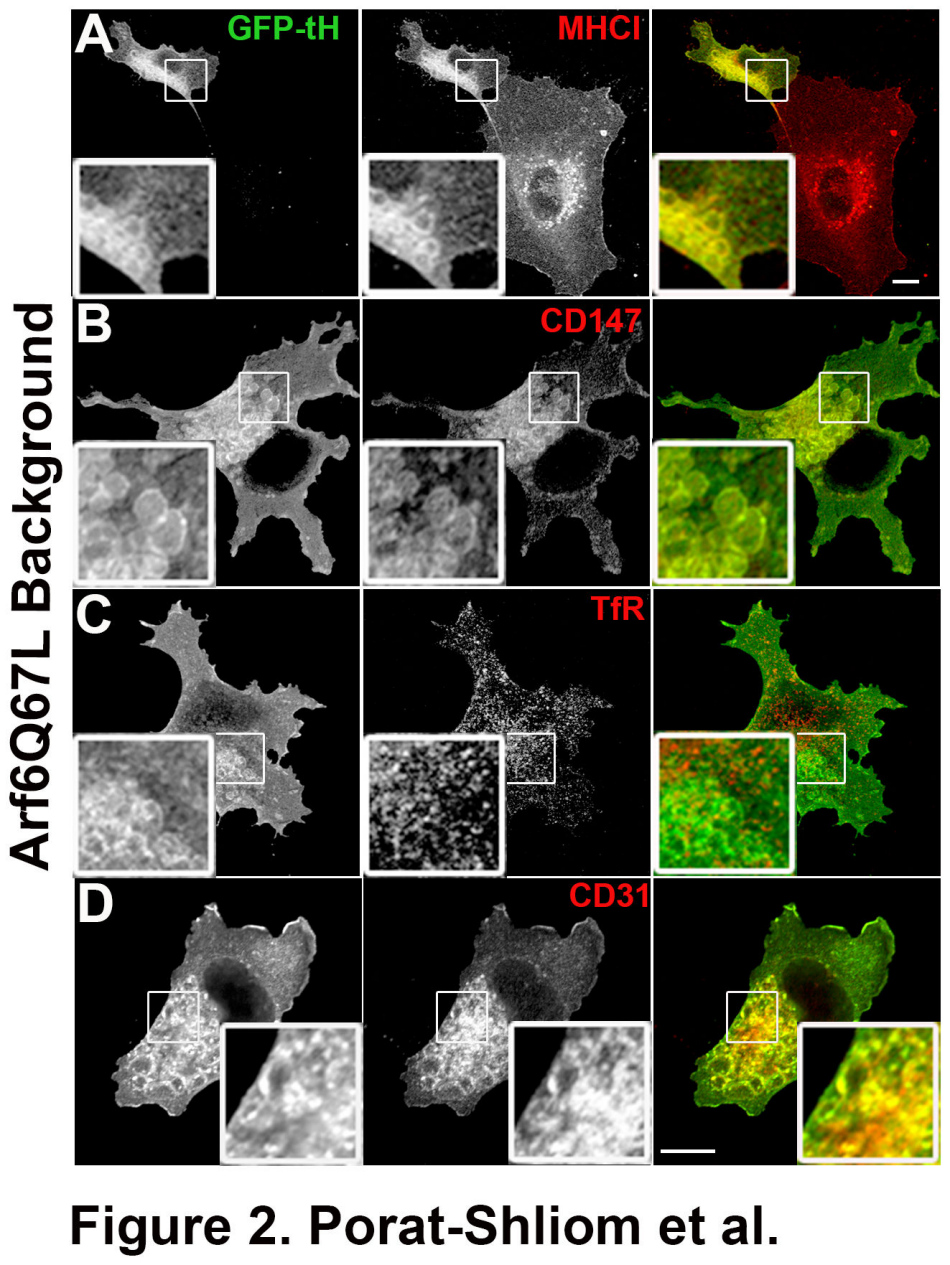
CIE, but not CME cargo proteins, accumulate in Arf6 Q67L vacuoles. Arf6Q67L was co-expressed with GFP-tH to form and label the vacuolar structures, respectively. Cells were labeled with antibodies against MHCI (A), CD147 (B), transferrin receptor (TfR) (C) or CD31 (D) using indirect Immunofluorescence as described in Materials and Methods. MHCI, CD147, and CD31 but not the TfR, were trapped in the Arf6 induced vacuolar structures. Bar, 10 μm.

Next, we examined the effect of expression of the constitutively active mutant of Arf6, Arf6Q67L, in HUVECs. Expression of Arf6Q67L in cells leads to the accumulation of enlarged vacuoles containing CIE cargo proteins. This mutant has been used as a diagnostic tool to determine if PM proteins traffic through CIE in other cell types [6]. H-Ras associates with CIE-derived endosomes and expression of the carboxyl terminal tail of H-Ras (referred to here as GFP-tH) was utilized for labeling the CIE pathway [21] and Arf6Q67L-induced vacuoles [10]. We observed that HUVECs tolerate expression of Arf6Q67L much better than other cell lines, which tend to round-up and detach from the surface (after 12 hours) due to continuous membrane internalization. We found that the Arf6Q67L-vacuoles were labeled with GFP-tH as shown in other cell types (Figure 2), and trapped MHCI (Figure 2A), CD147 (Figure 2B), and CD98 (not shown) but not the transferrin receptor (TfR) (Figure 2C). Interestingly, CD31 (PECAM), an endothelial marker that labels lumen membranes was also trapped in the Arf6Q67L vacuoles (Figure 2D). Taken together, these results demonstrate that CIE in HUVECs functions in a similar manner to that characterized in HeLa and COS cells. Furthermore, it suggests that CD31 might be a cargo molecule entering cells through CIE and that CIE endosomes that accumulate when the Arf6 Q67L mutant is expressed, might contribute membranes to the forming endothelial lumen. Supporting this idea is the fact that actin and β1-integrin are also enriched on the Arf6Q67L vacuoles [10] and label the newly formed endothelial lumens [43]. Interestingly, other groups studying endothelial lumen formation have used GFP-tH as a marker to label the newly formed lumen [37].

### β1-integrin Activating Antibody Induces the Formation of Enlarged Clathrin-Independent Endosomes

**Figure 3:**
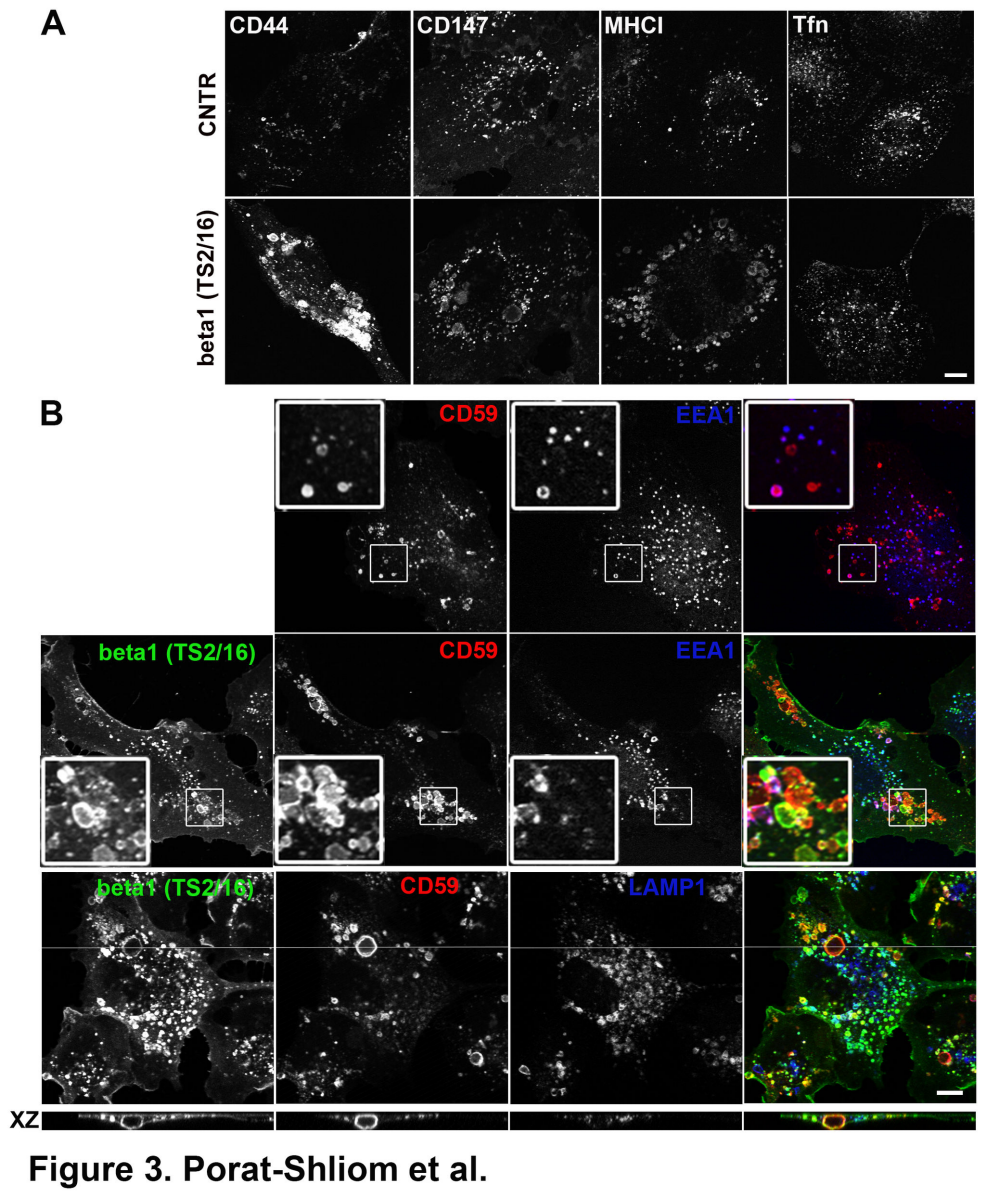
β1-integrin activating antibody induces the formation of enlarged clathrin-independent endosomes. (A) Internalization of fluorescently conjugated antibodies against CD44, CD147, MHCI or fluorescently labeled transferrin (Tfn) for 1 hour in the absence (upper panel) or presence (lower panel) of the β1-integrin activating antibody TS2/16. Note the change in endosome size in the presence of TS2/16 (compare upper and lower panels). Bar, 5 μm (B) Internalization of CD59 for 30 min in the absence (upper panel) or presence (middle and lower panels) of the β1-integrin activating antibody TS2/16. In the presence of TS2/16, CD59 was in enlarged endosomes that partially co-localized with EEA1 (see inset, middle) but did not co-localize with LAMP1 (lower panel). Side view (XY) demonstrating the enlarged endosome is positive for CD59 and TS2/16 but not LAMP1. Bar, 10 μm.

β1-integrin activating antibodies were shown to enhance endothelial lumen formation [44]. To further investigate the role CIE has in lumen formation we tested the effect of β1-integrin activating monoclonal antibodies (TS2/16) on CIE. Specifically, we tested whether TS2/16 can induce lumen-like structures in HUVECs grown on coverslips (2D), in the absence of collagen. We incubated fluorescently conjugated monoclonal antibodies against CD44, CD147 and MHCI or fluorescently labeled transferrin in the absence or presence of TS2/16. We found that following 60 min of internalization in the presence of TS2/16, clathrin-independent cargo proteins were present in large endosomes while transferrin-containing endosomes remained mostly unchanged (Figure 3A). Notably, the large endosomal structures were reminiscent of nascent lumens, observed in 3D matrices [43] and the vacuoles that form in cells expressing Arf6Q67L (Figure 2). 

To further learn about the nature of the enlarged endosomes, we followed the internalization of another CIE cargo protein and an apical membrane protein, CD59 [7] for 30 minutes in the absence or presence of TS2/16. In the absence of TS2/16, CD59 was present in scattered endosomal structures, some of which were also positive for EEA1 (Figure 3B upper panel). In the presence of TS2/16, we observed the formation of enlarged CD59 containing endosomes, which also partially co-localized with EEA1. Surprisingly, TS2/16 was present only in some of these enlarged endosomes (Figure 3B middle panel). Finally, the enlarged endosomes did not co-localize with LAMP1 (Figure 3B lower panel) suggesting these structures are not a degradative compartment and potentially represent a precursor intermediate for the endothelial lumen. We also followed internalization of clathrin-independent cargo proteins in the presence of TS2/16 for a period of 4 hours and found that the enlarged endosomes were no longer present in the cells (data not shown). Finally, in contrast to the findings with β1-integrin activating antibodies, the β1 or α2 blocking antibodies and the α2 activating antibodies had no obvious effect on CIE in HUVECs grown on coverslips (data not shown). Taken together, activating the β1-integrin in HUVECs grown in 2D induces the formation of enlarged endosomes that contain clathrin-independent cargo proteins. Like the Arf6Q67L vacuoles, they might share common features with nascent lumens however they are transient and most likely the membrane and content recycle back to the plasma membrane.

### MHCI and CD98 enter cells with fluorescent dextran in 3D collagen matrix

**Figure 4:**
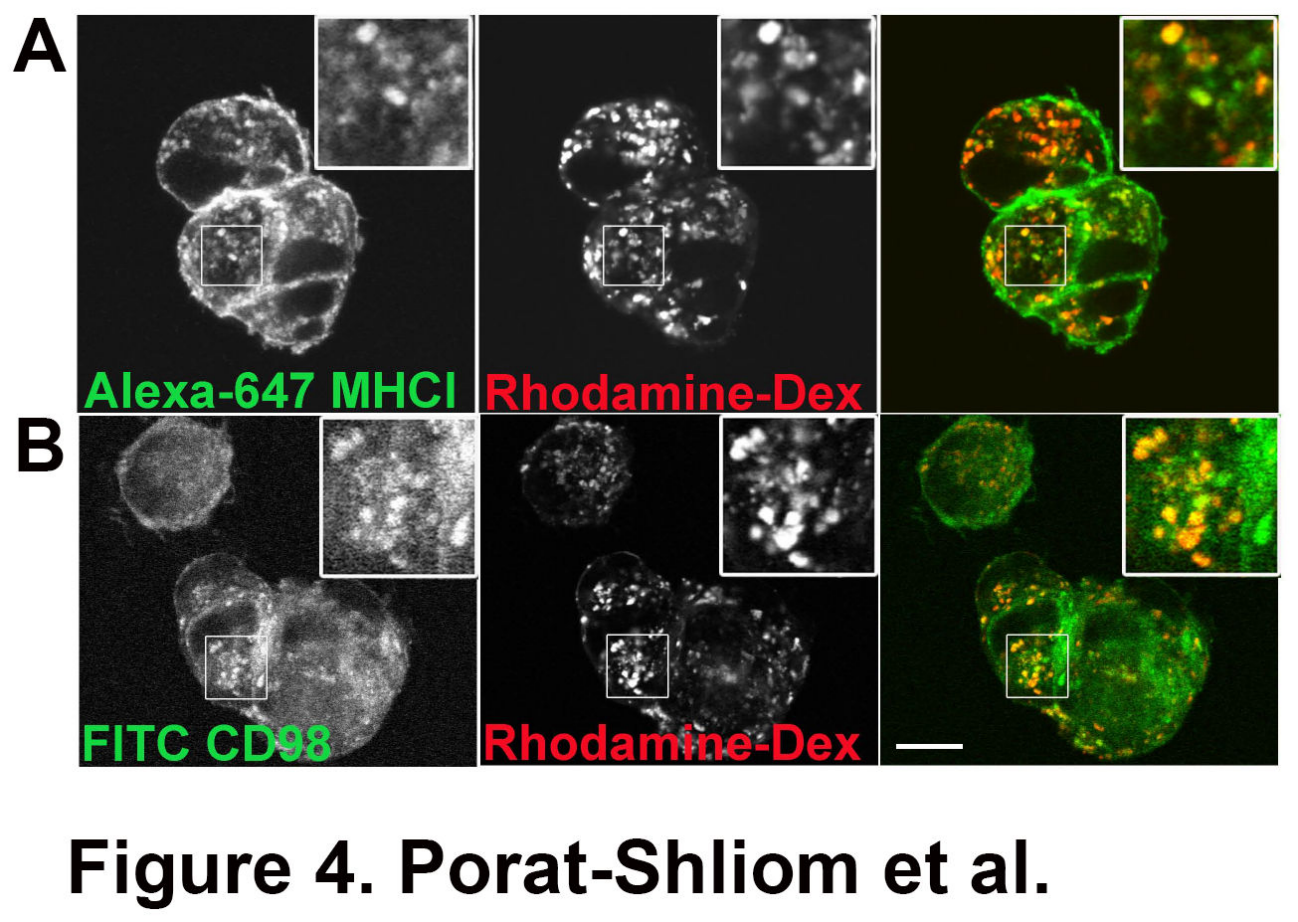
Internalized MHCI and CD98 co-localize with fluid phase markers in HUVECs. HUVECs were plated in three-dimensional collagen gels and allowed to internalize rhodamine-dextran and either Alexa-647 conjugated antibody against MHCI or FITC conjugated antibody against CD98 for 1 h. Cells were digested out of the collagen and plated onto poly-L-lysine coated coverslips. Pinocytic vesicles labeled with rhodamine dextran were obvious after 1 h of internalization and many of them were also positive for MHCI or CD98. Bar, 10 μm.

 When placed in three-dimensional collagen gels, HUVECs form a lumen in a process involving the internalization of pinocytic vesicles labeled with the fluid phase marker rhodamine dextran [43]. To examine whether HUVECs internalize fluid marker via CIE in 3D matrix, we cultured them in 1.5mg/ml collagen. After 1-hour incubation in a collagen gel with antibodies and dextran, the cells contained many internal endosomal structures that were labeled with the dextran fluid marker. These endosomes were also positive for MHCI and CD98, suggesting that these internalized endosomes were derived from CIE (Figure 4). 

### Nascent lumens contain MHCI but not transferrin receptor

**Figure 5:**
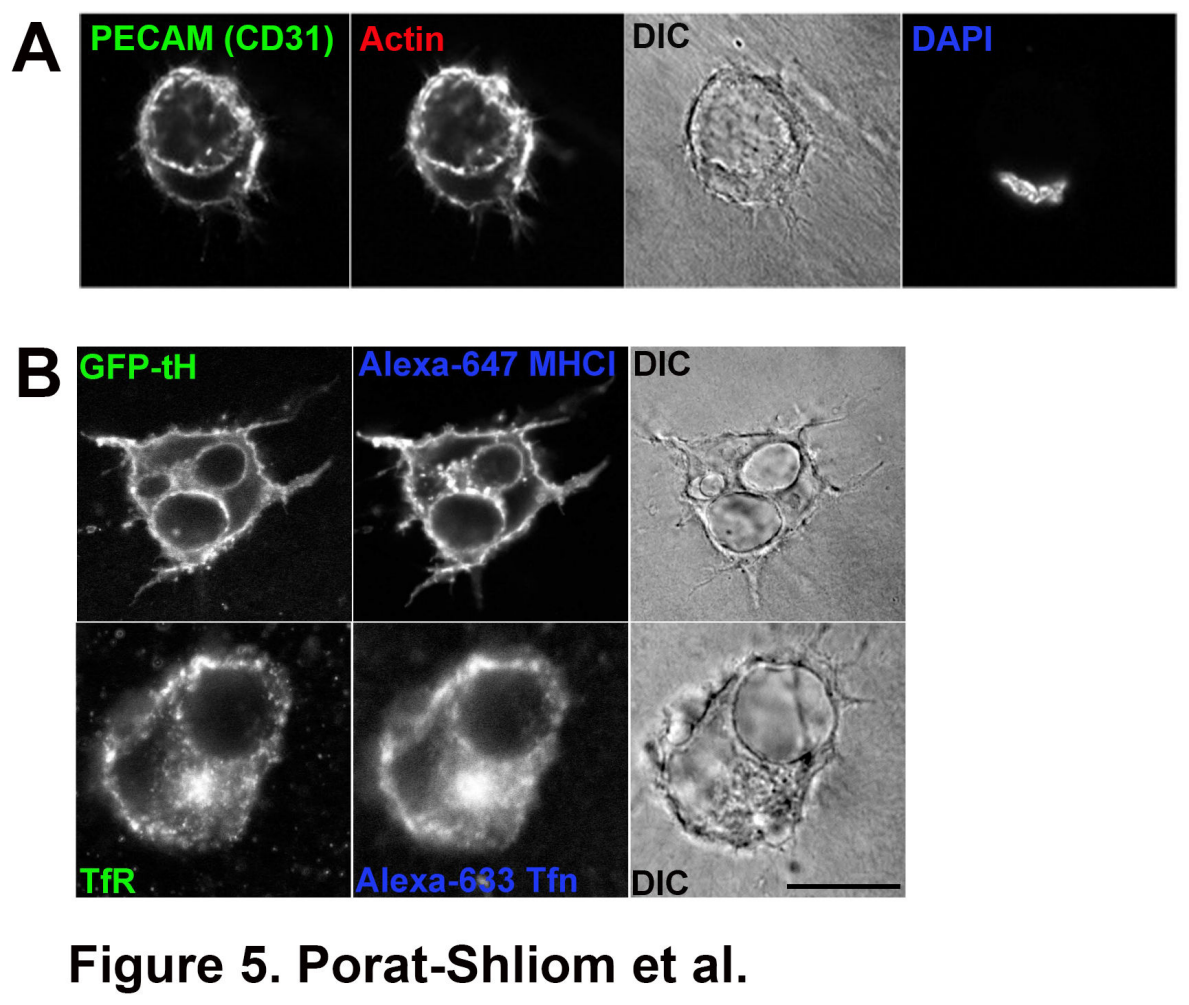
CIE cargo molecules label the newly formed endothelial lumen. (A) HUVECs were placed in three-dimensional collagen gels for 4 h to allow lumen formation. Newly formed lumens were identified using CD31 (PECAM) and rhodamine-phalloidin to label F-actin and DIC. (B) GFP-tH expressing or non-transfected cells were placed in three- dimensional collagen gels and allowed to internalize Alexa-647 conjugated antibodies against MHCI or Alexa-594 Tfn for 4 h. Cells were fixed and labeled with a polyclonal against GFP (B upper panel) and a monoclonal against the transferrin receptor (TfR) (B lower panel). MHCI was associated with the newly formed lumen membrane while internalized Tfn and the TfR were not. Bar, 10 μm.

 In order to examine the role CIE has in lumen formation and whether these unique membranes contribute to establishing and expanding the newly formed lumen, we followed internalization of CIE and CME cargo proteins in HUVECs cultured in collagen gels. We first tried to identify lumens in fixed preparations using indirect immunofluorescence using various lumen markers. As shown in Figure 5A, we were able to observe nascent lumens with rhodamine-phalloidin to label F-actin and antibody to CD31, a lumen marker. Only lumens that were clearly identified with the GFP-tH fluorescent marker or differential interference contrast microscopy (DIC) were imaged. Eighteen hours after electroporation of the plasmid encoding the lumen marker GFP-tH, cells were cultured in a three-dimensional collagen gel. Once the gels solidified, full media was added along with Alexa 647-conjugated antibodies against MHCI. The antibodies were allowed to internalize for 4 hours followed by fixation and mounting of the gels on slides. As seen in Figure 5B (upper panel), MHCI antibody was associated with the lumen membrane labeled with GFP-tH. Furthermore, smaller endosomal structures containing MHCI were scattered along the lumen membrane suggesting that endosomes containing CIE cargo proteins were fusing with the forming lumen (Figure 5B upper panel). Comparable results were obtained using antibody to CD147 (data not shown). To examine the trafficking of CME cargo, Alexa-594 transferrin was allowed to internalize for 4 hours and the cells were also labeled for the transferrin receptor using indirect immunofluorescence. The internalized transferrin and the receptor overlapped as expected, however, neither transferrin nor the transferrin receptor were present on the endothelial lumen (Figure 5B lower panel). This suggests that transferrin-containing endosomal membranes do not represent a major contributor of lumen formation.

## Discussion

Endothelial lumen formation is a complex developmental process that requires orchestrated environmental stimuli and cellular signaling. According to the 'cell hollowing' model accumulated pinocytic vesicles serve as the precursor for the newly formed apical pole, which will form the lumen [32]. A similar process was also observed *in vivo*, using zebrafish [37]. It is possible that endothelial cells, *in vivo*, at different stages of development and different environments will adopt different mechanisms of vasculogenesis [31-36]. Nevertheless, it is likely that endocytosis would be a main component in the generation, sorting and maintenance of the membranes forming the lumen.

 Although the signaling cascades involved in lumen formation have been extensively studied [32,43,45], very little is known about the endocytosis mechanism. Unlike CME, CIE internalizes and recycles membranes containing signaling molecules, lipids and cholesterol [3]. These signaling molecules are observed on the vacuoles accumulated upon the expression of Arf6Q67L. The vacuolar membranes have many common features with the plasma membrane, they contain phosphatidylinositol 4,5-bisphosphate, cholesterol and actin along with transmembrane proteins that traffic with CIE. Interestingly, fluid phase markers, GFP-tH and actin are used to label newly formed endothelial lumens. In addition β1-integrin is required and associates with the lumen [37,43]. This led us to hypothesize that CIE endosomes that accumulate when Arf6Q67L is expressed are the precursors for the forming lumen. In support of this we found that CIE is distinct from CME in HUVECs and that CIE cargo proteins accumulate in the vacuoles formed by Arf6Q67L (Figures 1 and 2). Additionally, we found that CD31, a frequently used marker for endothelial lumen, is trapped in the vacuoles supporting the possibility that CIE might have a role in this process. Indeed the Arf6Q67L vacuoles could represent the nascent apical membrane domains in cells grown in two-dimension containing many PM proteins, lipids and signaling molecules necessary for lumen formation. 

 Finally, we observed that the β1 integrin activating antibody, TS2/16, induces the formation of enlarged endosomes in HUVECs grown in 2D. Indeed the β1-integrin activating antibody was shown to enhance lumen formation in 3D while the blocking antibody abolished the process [43,44]. More recently, β1-integrin was shown to be essential for the establishment of endothelial cell polarity *in vivo* [46]. These enlarged endosomes contain an array of cargo proteins that enter cells by CIE, however unlike the Arf6Q67L vacuoles that block further trafficking of these proteins, these enlarged endosomes are transient and most likely recycle back to the plasma membrane without the additional signaling required for lumen formation and maintenance. Interestingly, both apical (CD44, CD59) and basolateral (MHCI, CD147, CD98) proteins are internalized via CIE and reach the nascent lumens both in 2D (Figures 2 and 3) and 3D (Figures 4 and 5) suggesting further sorting events are required for proper targeting of these proteins.

Additional experiments using the three-dimensional collagen culture system gave further support to the role of CIE in lumen formation. We found that both fluid markers and CIE cargo proteins (MHCI and CD98), localized to the newly formed lumen (Figure 4 and 5). Furthermore, MHCI endosomes were observed adjacent to the lumen membrane supporting our hypothesis that CIE and delivery of CIE cargo proteins to the lumen is involved in endothelial lumen formation. Another CIE cargo protein, CD147 was associated with Arf6Q67L vacuoles (Figure 2) and the lumen (not shown). CD147 is also known as extracellular matrix metalloproteinase inducer (EMMPRIN) and was shown to be over-expressed in many types of cancer and to induce MMPs activity [47,48]. This is intriguing in the context of endothelial lumen formation since MT1-MMP was shown to be required for lumen formation [49]. Finally, an additional CIE cargo protein, CD98 was shown to interact with β1-integrin affecting epithelial branching morphogenesis [50]. This might also suggest that aside for the cargo proteins, other signaling molecules associated with the CIE pathway, may be involved in the lumen formation process and that their targeting to the newly formed apical domain is required for further development of a functional vasculature. Specifically, it would be of interest to test the role that Arf6 has in lumen formation primarily since constitutive activation of Arf6 leads to the formation of vacuoles that have many common features with the endothelial lumen. Furthermore, it has been reported that Arf6 is a key player in epithelial cyst polarity [40]. Unfortunately, our preliminary experiments showed that there are some viability issues using the 3D collagen essay with transfected HUVECs (not shown). 

The transferrin receptor was absent from the lumen membrane (Figure 5) and consistent with this, it has been reported that clathrin is absent from the newly formed endothelial lumen whereas caveolin is present [43]. Furthermore, we noticed that endosomes that contained the transferrin receptor and lysosomes were not adjacent to the newly formed lumens but were scattered throughout the cell (unpublished observations); this contrasted with the MHCI endosomes that concentrated on and around the lumen (Figure 5). Although the transferrin receptor was not present on the lumens, this does not prove that CME input does not contribute to lumen formation. Further experiments are required to evaluate what role CME plays in endothelial lumen formation. Nevertheless, the results that the Arf6Q67L vacuoles have many common features with endothelial lumens support the idea that incoming endosomes containing CIE cargo proteins are important contributors to the formation of the endothelial lumen. 

Our findings suggest a role for CIE in lumen formation, however further investigations are required to reveal the underlying mechanism. Identifying common regulators that participate in both the membrane trafficking and this morphogenic process would allow us to either inhibit or accelerate lumen formation. Given the prominent role caveolae play in transcytosis across mature endothelial cells, it will be important to examine whether caveolar proteins are involved in the initial internalization. In conclusion, this study reveals a novel function for non-canonical endosomes loaded by CIE: delivery of membranes, cargo proteins and potentially lipid and signaling molecules to nascent lumens. Hence, endosomes derived from CIE contribute to the achievement and maintenance of cell polarity.

## Materials and Methods

### Reagents and Antibodies

Rabbit polyclonal antibody to Arf6 [51] and mouse monoclonal antibody (mAb) to human MHCI (W6/32) [6] were described previously. Mouse monoclonal anti-early endosomal antigen 1 (EEA1) was purchased from BD Biosciences (Palo Alto, CA). Rabbit anti-Lamp1 and Rabbit polyclonal anti-CD31 were purchased from Abcam (Cambridge, MA). Rabbit polyclonal antibody to EEA1 was purchased from BD Biosciences (Palo Alto, CA). Invitrogen (Carlsbad, CA) was the source for transferrin (Tfn) conjugated to Alexa-633 and Alexa-594, rabbit polyclonal antibody to GFP, rhodamine-dextran and all secondary antibodies conjugated to Alexa-594, -488, and -647. Mouse monoclonal to human MHCI (w6/32), CD98 (MEM-108), CD147 (HIM6), CD59 (H19), CD29 (TS2/16), Alexa-647 conjugated antibodies against MHCI (w6/32), Alexa-647 conjugated antibodies against CD147 (HIM6), FITC conjugated antibodies against CD98 (MEM-108), Alexa-488 conjugated antibodies against CD29 (TS2/16), Alexa-488 conjugated antibodies against CD44 (IM7) were purchased from Biolegend (San-Diego, CA). Blocking antibodies against CD29 (Mab13) were purchased from BD Biosciences (San Jose, CA). All other reagents were purchased form Sigma-Aldrich (St. Louis, MO).

### Cell culture

HUVECs were purchased from LONZA (Walkerrsville Inc.) and grown in flasks coated with 30μg/ml human fibronectin (Sigma St Louis, MO) in Endothelial Cell Growth Medium-2 (EGM-2, Lonza Walkerrsville Inc.) at 37°C with 5% CO_2_. HUVECs were grown for approximately 25 passages. For experiments, cells were plated either on coverslips coated with fibronectin or in collagen gels (see below).

### Three-dimensional collagen system

The preparation of the collagen gels was adopted from the protocol provided by BD Biosciences and modified according to the system requirements. Rat-tail collagen was purchased from Millipore (Billerica, MA) and gels were made as follows. For the preparation of 1 ml collagen mixture at a final concentration of 1.5 mg/ml, the volume of collagen to be added was calculated according to the stock concentration. Microcentrifuge tubes were pre-cooled on ice and 100 μl of cold 10X M199 media was added. Sodium hydroxide (NaOH) was used to neutralize the pH. The volume added to the collagen mixture was calculated using the following formula: (volume of collagen to be added) x 0.023 ml = volvolume 1 Molar NaOH. Cold 1X M199 media was used to complete the volume to 1 ml. The mixture was stored on ice until used. Cells were added to the mixture at a concentration of 2x10^6^ in 1ml. The mixture was then distributed into a 96-well plate (28μl per well) that was placed in a tissue culture incubator (37°C with 5% CO2) to solidify for 30 min followed by the addition of EGM2-containing 80 nM phorbol 12-myristate 13-acetate (PMA). When internalization assays were performed during lumen formation, the media also contained Alexa-647 conjugated antibody against MHCI, Alexa-647 conjugated antibody against CD147, FITC conjugated antibody against CD98, Alexa-633 transferrin or rhodamine dextran, as indicated. Cells were incubated in 37°C with 5% CO_2_ to allow lumens to develop for the indicated time. Samples were either processed for indirect immunofluorescence or digested out of the collagen using collagenase (Sigma St Louis, MO) at 37°C and plated on poly-L-lysine coated coverslips.

### Plasmids and Transfection

Arf6Q67L was in pXS plasmid (Radhakrishna and Donaldson, 1997). GFP-tH encoding GFP fused to the double palmitoylated and farnesylated carboxy terminal tail of H-Ras was from Clontech (Mountain View, CA). Electroporation of HUVECs was performed according to the manufacturer (Amaxa). Briefly, cells were trypsinzed, mixed with the recommended nucleofection solution together with 2µg DNA and electroporated using the optimized program. Following electroporation, HUVECs were plated on fibronectin for an 18 hours recovery before placing them in a collagen gel.

### Immunofluorescence

For endocytosis of transferrin and antibodies against MHCI, cells were serum starved for 30 min at 37°C in DMEM alone, and then 5 μg/ml fluorescently labeled transferrin and/or 0.01 μg/ml monoclonal antibodies against cargo proteins were added. Cells were then incubated for the time indicated at 37°C to allow endocytosis. For NH_4_Cl treatment, cells were transferred to full media containing 25 nM NH_4_Cl for approximately 14 h. At the end of incubation, PM-associated ligand and antibodies were removed by rinsing the cells in low pH solution (0.5% acetic acid and 0.5 M NaCl, pH 3.0) for 20–30 seconds. Cells were then fixed with 2% formaldehyde in phosphate-buffered saline (PBS) at room temperature for 10 min. Internalized antibodies directed to CIE cargo proteins were labeled with fluorescently conjugated (i.e. Alexa-488, -594 or -633) secondary antibodies in the presence of 0.2% saponin. For internalization in the presence of TS2/16, fluorescently conjugated (i.e. Alexa-488 or FITC) antibodies against CD44, CD147, MHCI or fluorescently labeled transferrin were internalized in the presence or absence of unlabeled TS2/16 for 60 min followed by the removal of plasma membrane bound antibodies using low pH solution and fixation. For indirect immunofluorescence, cells were fixed as described above and immunostained as described previously (Naslavsky et al., 2003). Briefly, after fixation cells were incubated for 1 h with primary antibody diluted in 10% fetal calf serum in PBS in the presence of 0.2% saponin. After washing, cells were incubated for 1 h with secondary antibody diluted as described above. All images were obtained using a 510 LSM confocal microscope (Carl Zeiss, Thornwood, NY) with 63 x 1.3 numerical aperture PlanApo objective. After acquisition, images were handled using Adobe Photoshop (Adobe Systems, San Jose, CA). ImageJ (National Institute of Health) was used for co-localization analysis. Images were manually thresholded and an image containing the overlapping pixels (i.e. CD98 and EEA1 or MHCI and EEA1) was generated using the multiply feature in the image calculator function. Finally, Individual cells were outlined, and the overlapping integrated fluorescence intensity was divided by the integrated fluorescence intensity of either MHCI or CD98 to calculate percent co-localization. All experiments were confirmed at least three times, and a representative image is shown.

## Supporting Information

Figure S1 **MHCI and CD98 share trafficking itinerary in HUVECs.** (A) Percent co-localization of CD98 (mean: 19.93±10.7; n=13 cells) and MHCI (mean: 46.49±16.89; n=5 cells) with EEA1 after 30 min of internalization was analyzed as described in Materials and Methods. Representative image with selected cells for analysis is presented (left side). **Statistical significance at p<0.02 was calculated using unpaired t-test. (B) MHCI and CD98 were internalized in the presence of NH_4_Cl to inhibit lysosomal degradation. Cells were processed for immunofluorescence 24 h later and stained with LAMP1 to label lysosomes. Both MHCI and CD98 were observed in lysosomes. Bar, 5 μm.
(TIF)
