# Supplementary figures and images for: Endosomes Derived from Clathrin-Independent Endocytosis Serve as Precursors for Endothelial Lumen Formation
Source: PLoS One. 2013 Nov 25;8(11):e81987. doi: 10.1371/journal.pone.0081987 (PMC3839925; doi:10.1371/journal.pone.0081987)

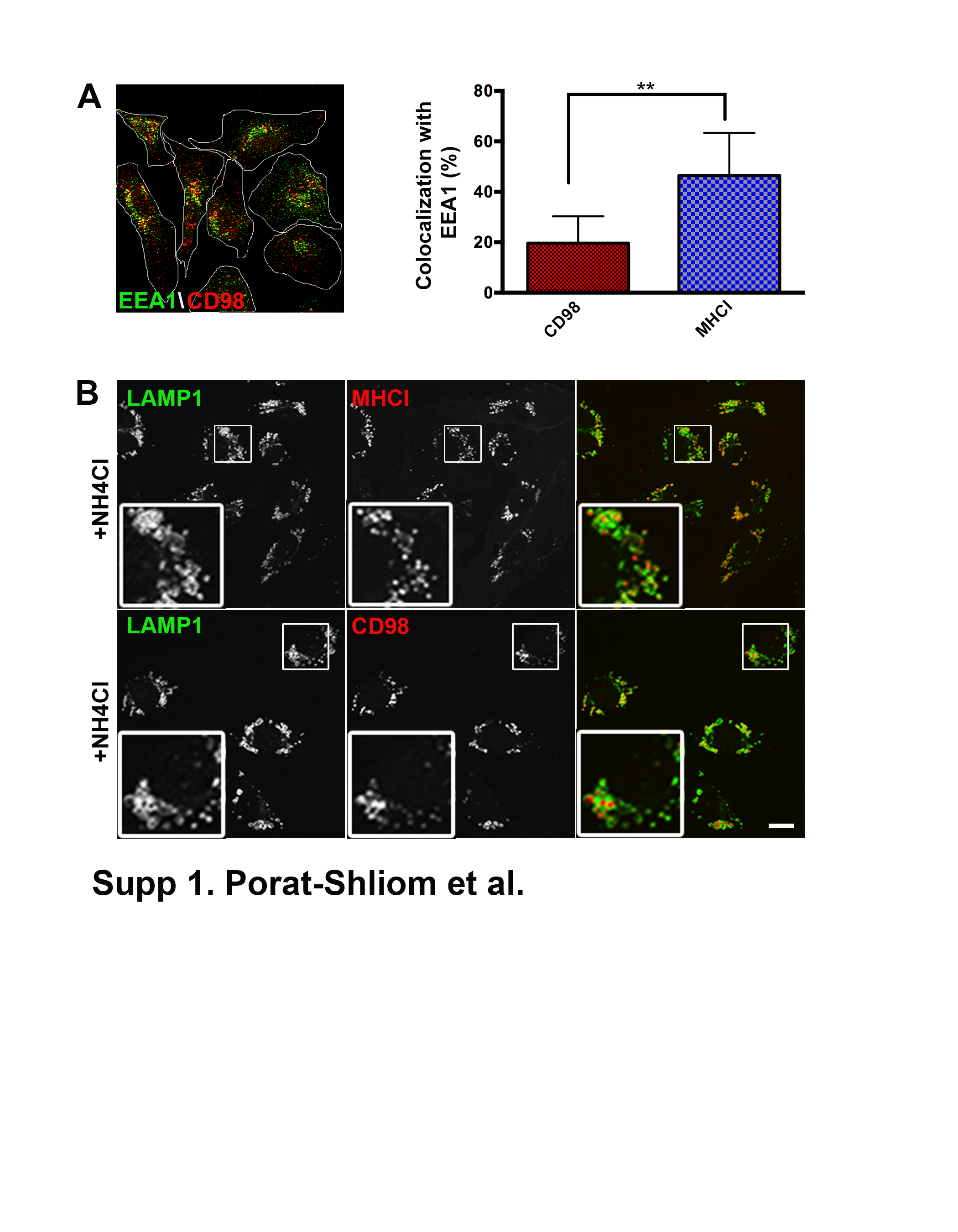

Supplement: Figure S1 — MHCI and CD98 share trafficking itinerary in HUVECs. (A) Percent co-localization of CD98 (mean: 19.93±10.7; n=13 cells) and MHCI (mean: 46.49±16.89; n=5 cells) with EEA1 after 30 min of internalization was analyzed as described in Materials and Methods. Representative image with selected cells for analysis is presented (left side). **Statistical significance at p<0.02 was calculated using unpaired t-test. (B) MHCI and CD98 were internalized in the presence of NH4Cl to inhibit lysosomal degradation. Cells were processed for immunofluorescence 24 h later and stained with LAMP1 to label lysosomes. Both MHCI and CD98 were observed in lysosomes. Bar, 5 μm. (TIF) [file pone.0081987.s001.tif]
